# Prevalence of eating disorder symptomatology among outpatients referred to health promotion from somatic hospital departments

**DOI:** 10.1186/s12888-023-05331-5

**Published:** 2023-11-15

**Authors:** Signe Graungaard, Tobias Lund Christensen, Lise Noerregaard Soendergaard, Gry Kjaersdam Telléus

**Affiliations:** 1 Department of Health Promotion, Aalborg University Hospital, Hobrovej 18-22, Aalborg, 9000 Denmark; 2 Unit for Psychiatric Research, Aalborg University Hospital, Mølleparkvej 10, Aalborg, 9000 Denmark; 3 Institute of Communication and Psychology, Psychology, Aalborg University, Aalborg, Denmark

**Keywords:** Eating disorders, Obesity, People having larger bodies, Binge eating disorder, Grazing

## Abstract

**Background and Aims:**

All eating disorders (EDs) lead to a significant decrease of health status, psychosocial functioning and quality of life (QoL). Individuals with untreated binge eating disorder (BED) tend to gain weight over time, which may contribute to serious health issues. In somatic hospital departments, some outpatients have reduced compliance with lifestyle changes. This may, to some extent, be due to patients with an undiagnosed ED receiving the incorrect treatment. In this cross-sectional study, we aimed to investigate the prevalence of EDs among patients referred to lifestyle courses.

**Results:**

A total of 136 patients referred from somatic hospital departments to lifestyle changes in a specialized hospital unit were included in the study. The response rate was 69.4%. Self-reported ED or sub-clinical symptoms of ED according to the Eating Disorder Examination Questionnaire (EDE-Q) were found in 17.65%. Of these, 11.03% fulfilled the self-reported criteria for an ED (BED, 7.35%; bulimia nervosa, 3.68%). Patients with an ED or subclinical ED symptoms had elevated grazing behaviour compared to those without ED symptomatology. A statistically significant difference in QoL was also found.

**Discussion and Conclusions:**

The prevalence of self-reported ED or subclinical ED symptoms in patients referred to a lifestyle course is substantial. This ED group had reduced QoL and larger grazing behaviour compared to patients without ED symptomatology. Thus, the prevalence of undiagnosed EDs among patients within somatic hospital departments may be substantial, underlining the importance of screening and further research within this topic.

**Level of Evidence:**

Level III, well-designed cohort study.

**Significance:**

What is already known on this subject? In a review including populations from Scandinavia, the USA and South America, the estimated BED prevalence in individuals with higher body weight seeking help to lose weight is 13–27% [22]. Dawes et al. (2016) conducted a meta-analysis investigating the prevalence of mental health conditions among bariatric surgery candidates and recipients. They included 25 studies with a total of 13,769 patients and found that the prevalence of BED was 17% (13–21%) [10]. What this study adds? We have identified a group of patients who may be receiving inappropriate treatment with weight loss intervention instead of specialized ED intervention. It appears that this issue is valid in various somatic hospital departments. Thus, this is a field that requires further attention and investigation.

## Background

Eating disorders (EDs) lead to a significant decrease of health, psychosocial functioning and quality of life (QoL) [1, 2]. Individuals with untreated binge eating disorder (BED) tend to gain weight over time, which can lead to serious health issues such as diabetes and other metabolic dysfunctional conditions (McCuen-Wurst et al., 2018). Increased psychopathology is also seen in patients with BED, including anxiety, depression and mood, etc. [3, 4]. Grazing is characterized by uncontrolled repetitive eating of smaller amounts of food. Grazing behaviour is associated with several ED symptoms, more severe ED psychopathology, higher body mass index (BMI) greater psychological distress and lower mental health-related QoL [5–7].

A large study conducted in 14 European countries with a total of 24,124 participants showed that only 38.3% of individuals diagnosed with BED received treatment. Similarly, only 47.4% of individuals diagnosed with bulimia nervosa received treatment for the condition during their lifetime [8]. In a study from the UK with 5658 participating women between 40 and 50 years of age, only 27.4% of the women with an ED sought help or received treatment for their ED during their lifetime [9]. This may be due to a combination of several factors, such as lack of screening, information or personal resources.

Dawes et al. (2016) conducted a meta-analysis investigating the prevalence of mental health conditions among bariatric surgery candidates and recipients. They included 25 studies and investigated the prevalence of BED in a total of 13,769 patients. The BED prevalence was 17% (13–21%) [10].

In the Department of Health Promotion at Aalborg University Hospital, Denmark, we experience that some patients have reduced compliance with lifestyle changes, and we suspect that this may be due to undiagnosed EDs and consequent incorrect treatment.

We hypothesized that EDs would occur in individuals referred to weight loss intervention in the somatic hospital.

### Aim

In this cross-sectional study, we aimed to investigate the prevalence of EDs among patients referred to lifestyle courses at the Department of Health Promotion, Aalborg University Hospital, Denmark. Furthermore, we aimed to investigate if patients with an ED had increased grazing behaviour and decreased QoL.

## Methods

This study was conducted as a cross-sectional study based on patient-reported questionnaires and additional data from medical records. All patients following the lifestyle course at the Department of Health Promotion, Aalborg University Hospital, Denmark, were invited to participate in the study. The Department of Health Promotion at Aalborg University Hospital is a unique department and not a standard part of the Danish hospital structure. The department aims at health promotion with regard to diet, smoking, alcohol and exercise. The main intervention at the department is based on a lifestyle course conducted by a professional nutritionist. The course consists of individual consultations. The frequency of consultations and the duration of the course are individual but for the majority it consists of 8–12 consultations within 12 months; however, in some cases this will be followed by an additional 12 months of monitoring. Patients are referred from all the somatic departments at the University Hospital. Patients with a BMI > 27 who are waiting for or undergoing treatment in the hospital and where weight loss is important for the patient’s further treatment can get referred to the lifestyle course. Furthermore, the patient must be motivated for a lifestyle change. Referral may be to prevent disease relapse and the reduction or prevention of further comorbidities but weight loss may be a condition for patients to receive treatment (e.g. before possible surgery). The primary goal is lifestyle change regarding eating and exercise habits aiming to improve overall somatic health state. Patients are referred from all hospital departments, thereby constituting a mixed group. Patients were initially referred to the various somatic departments from their general practitioner for various reasons, such as sleep apnoea, elevated liver count, in vitro fertilization (IVF), increased intracranial pressure, diabetes type II, inflammatory bowel disease and suppurating hidrosadenitis, and patients may have several comorbid conditions.

### Sample

All patients who were referred to the lifestyle course at the department and met the inclusion criteria were invited to participate in the study. Patients were consecutively included by phone and the data were collected between September 2022 and March 2023. Patients already following the intervention as well as patients consecutively referred for intervention were invited to participate 2 weeks after their initial consultation at the department. To be included in the study, patients should be at least 18 years old, able to read and understand Danish and able to receive electronic mail in the official Danish electronic mailbox system (e-Boks). Patients were contacted by phone, with a maximum of four calls at different times and dates. If it was not possible to reach patients by the fourth call, no further action was taken and the patient was excluded from the study. If agreeing to participate in the study, the patient would receive an additional e-Boks message with further information regarding the study. The invitation included a hyperlink to a questionnaire that could be completed in a web browser at home 3 weeks after the initial invitation; reminders were sent to those who had not yet completed the questionnaire.

### Assessment

Research Electronic Data Capture (REDCap) [11, 12] was used for data collection and data management. REDCap is a worldwide online system developed specifically for non-commercial clinical research. It is used for creating and managing databases and online questionnaires for research use [11, 12]. REDCap is used as default in clinical research at hospitals in Denmark. The self-reported questionnaire was designed by including demographic items and the instruments mentioned below. Demographic items were self-reported and included age, gender, height and weight, civil status, employment status and education level.

### Eating disorder

The Eating Disorder Examination Questionnaire (EDE-Q) [13, 14] is a widely used self-reported questionnaire that assesses the range and severity of ED behaviours based on the DSM-V manual. It is a 28-item questionnaire that includes a global score and four subscales of underlying psychopathology (restraint; eating concern; shape concern; weight concern). Most items are rated on a seven-point Likert scale, with scoring in the range 0–6. Scores are summed to obtain a global score, with a higher score indicating more severe ED behaviours. Subscale scores are obtained by adding the ratings for the relevant items and dividing by the total number of items in the specific subscale. By following different criteria, the EDE-Q can determine specific EDs in the sample [13, 14]. Measures of the EDE-Q constituted the primary outcome of this study and we used the Danish validated version [15, 16].

### Grazing

The Grazing Questionnaire (GQ) [17] measures behaviours and cognitions related to grazing and includes eight items. Five items assess eating behaviours and three items assess cognitions concerning loss of control while ‘grazing’. Items are rated on a five-point Likert rating scale over the range 0–4 and scores are summed to create a total score. Grazing behaviours and cognitions are better represented by higher scores [17]. The GQ was translated for use in this study. The GQ was first translated by two independent translators, then consensus translated, and finally back-translated by a third bilingual professional.

### Quality of life

The validated Danish version of EuroQol’s EQ-5D-5 L questionnaire [18, 19] was used to measure QoL and has five dimensions: mobility, self-care, usual activities, pain/discomfort and anxiety/depression. Every dimension has five levels, from no problems to extreme problems, and is given a one-digit number between 1 and 5. The patient’s health state is a five-digit score that combines the five dimensions. In addition, the EQ-5D-5 L contains a visual analogue scale (VAS) that measures self-rated health on a scale of 0–100, from ‘The best health you can imagine’ to ‘The worst health you can imagine’ [18, 19].

### Binge eating disorder

The Binge Eating Disorder Questionnaire (BED-Q) (Jensen et al., 2020) is a new Danish BED scale that addresses ED behaviours in the last three months. The BED-Q includes nine items that are rated on a five-point Likert rating scale, with scoring in the range 0–5. Scores are summed to produce a binge eating stress score. No symptoms of binge eating are equivalent to zero and a higher score represents more severe binge eating symptoms [20].

### Statistical analysis

STATA/MP 17.0 for Windows was used to perform the statistical analysis. Patients who only partially completed the EDE-Q were excluded from analysis. For descriptive statistics, the number of filled-in replies (*N*) and percentage (%) or the mean ± standard deviation (SD) were presented. BMI was categorized according to the World Health Organization (WHO) classification [21]. Linear regression adjusted for sex, age and BMI was used to compare the means of ED + and ED- on all the main outcomes of the used questionnaires. Assumptions for normality and variance homogeneity were investigated visually. A significance level of 0.05 was used (*p* < 0.05).

## Results

**Fig. 1:**
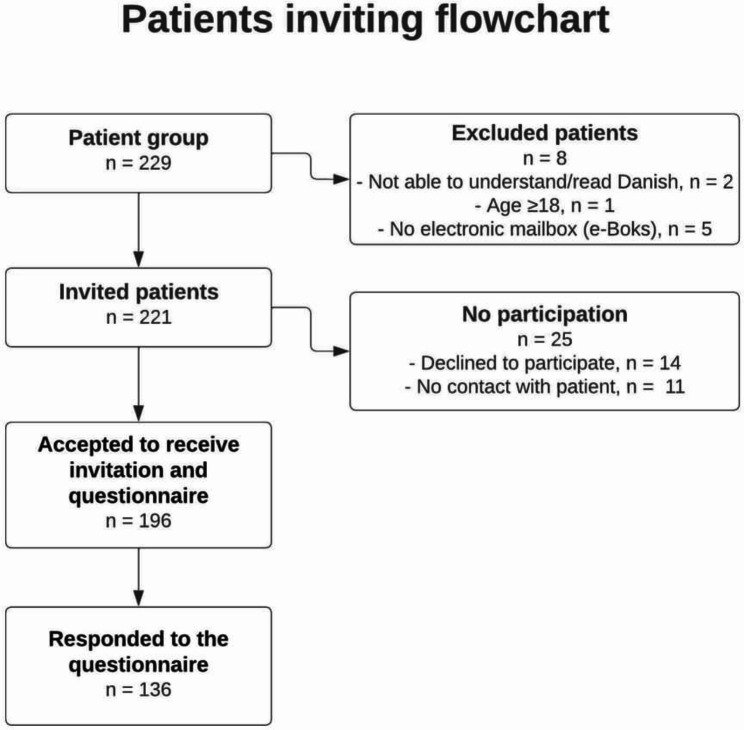
Flowchart of patients

A total of 229 patients were referred for intervention during the inclusion period. Eight patients did not meet the inclusion criteria and were excluded from the study. Fourteen patients were successfully contacted by phone but did not wish to participate in the study and 11 patients ware not possible to reach patients by the fourth call and therefore they were not included in the study. A total of 221 patients were considered eligible and invited for study. Of the 196 patients who accepted the invitation, 136 completed the questionnaire, yielding a response rate of 69.4% (Fig. 1).

### Characteristics of the sample

Patients included in the study were, on average, 46.57 (± 15.66) years old and had an average BMI of 36.62 (± 6.28) kg/m^2^. A total of 88 patients (64.71%) were assigned female at birth while 47 (34.56%) were assigned male at birth. In terms of level of education, 41.04% had completed a vocational education or short higher education course, 40.30% had completed a medium or long higher education programme and 18.66% had completed high school or college. Approximately half of the included patients (54.48%) were employed, 23.13% were either retired or on early retirement, 11.19% were studying, 8.96% were unemployed and < 5 patients were on sick leave. The 88 patients (64.71%) assigned female at birth were, on average, 44.06 (± 16.06) years old with an average BMI of 37.01 (± 6.49) kg/m^2^. Among patients assigned male at birth, the average age was 51.72 years with an average BMI of 36.00 (± 5.89) kg/m^2^. Table 1 shows the demographic information. Data on ethnic origin are not reported due to the risk of microdata (< 5 patients had a background other than ethnic Danish). Denmark is a highly homogeneous country in terms of ethnic background and the study population does not deviate from this.

**Table 1:** Demographics

Variables	All N = 136 (100%)	Assigned female at birth N = 88 (64.71%)	Assigned male at birth N = 47 (34.56%)
Age, mean years (SD)	46.57 (± 15.66)	44.06 (± 16.06)	51.72 (± 13.53)
Age category (years), *n* (%)			
18–29	26 (19.12%)	21 (23.86%)	< 5
30–39	23 (16.91%)	20 (22.73%)	< 5
40–49	23 (16.91%)	11 (12.50%)	12 (25.53%)
50–59	30 (22.06%)	13 (14.77%)	17 (36.17%)
60+	34 (25.00%)	23 (26.14%)	11 (23.40%)
BMI, mean kg/m^2^ (SD)	36.62 (± 6.28)	37.01 (± 6.49)	36.00 (± 5.89)
BMI category, *n* (%)			
25.0–29.9 kg/m^2^	20 (14.71%)	11 (12.50%)	9 (19.15%)
30.0–34.9 kg/m^2^	41 (30.15%)	26 (29.55%)	14 (29.79%)
35.0–39.9 kg/m^2^	40 (29.41%)	27 (30.68%)	13 (27.66%)
40 kg/m^2^	35 (25.74%)	24 (27.27%)	11 (23.40%)
Occupational status, *n* (%) (*N* = 134)			
Employed	73 (54.48%)	48 (54.55%)	25 (55.56%)
Unemployed	12 (8.96%)	9 (10.23%)	< 5
Students	15 (11.19%)	11 (12.50%)	< 5
Sick leave	< 5	< 5	< 5
Retired/early retirement	31 (23.13%)	18 (20.45%)	13 (28.89%)
Education level, *n* (%) (*N* = 134)			
High school or college	25 (18.66%)	21 (23.86%)	< 5
Vocational education or short higher education	55 (41.04%)	32 (36.36%)	22 (48.89%)
Medium or long higher education	54 (40.30%)	35 (39.77%)	19 (42.22%)

*Intersex is not reported due to microdata. SD = standard deviation, BMI = body mass index

### Prevalence of eating disorders in the sample

Less than one-fifth (17.65%) of patients had a self-reported ED or subclinical ED symptoms (henceforth referred to as ED+) according to the EDE-Q. Of the 11.03% who met the criteria for a self-reported ED, 7.35% were classified with BED, 3.68% met the criteria for self-reported bulimia nervosa and while 6.62% had subclinical ED symptoms. The EDE-Q global score was 3.38 (± 1.13) for patients with ED + using a self-reported questionnaire and 2.02 (± 0.99) for patients not demonstrating an ED (henceforth referred to as ED−). There was a significant difference between the two subgroups on the EDE-Q global score (*p* < 0.001) and for all subscales: restraint (p = 0.009), eating concern (p < 0.001), shape concern (p < 0.001), and weight concern (p < 0.001). Data regarding the EDE-Q are reported in Table 2.

**Table 2:** The Eating Disorder Examination Questionnaire (EDE-Q)

Variables	All *N* = 136 (100%)		
EDs, *n* (%)			
No ED (ED−)	112 (82.35%)		
All EDs & subclinical (ED+)	24 (17.65%)		
All EDs	15 (11.03%)		
BED	10 (7.35%)		
Bulimia & atypical bulimia*	5 (3.68%)		
Subclinical	9 (6.62%)		
	**ED+*N*** **= 24 (17.65%)**	**ED**− ***N*** **= 112 (82.35%)**	**Mean difference [95% CI]**, ***p***
EDE-Q Global Score, mean score (SD)	3.38 (± 1.13)	2.02 (± 0.99)	1.20 [0.75, 1.64] *p* < 0.001
EDE-Q subscales, mean score (SD)			
Restraint	2.75 (± 1.69)	1.96 (± 1.23)	0.81 [0.20, 1.41] *p* = 0.009
Eating concern	2.51 (± 1.33)	0.71 (± 0.99)	1.66 [1.19, 2.31] *p* < 0.001 1.16 [0.54, 1.78]
Shape concern	4.16 (± 1.35)	2.72 (± 1.46)	*p* < 0.001 1.15 [0.65, 1.64]
Weight concern	4.11 (± 1.16)	2.69 (± 1.19)	*p* < 0.001

*Bulimia and atypical bulimia are combined due to microdata. The means of the groups are compared using linear regression adjusted for sex, age and BMI. ED = eating disorder, BED = binge eating disorder, CI = confidence interval

### Eating disorder symptomatology

As displayed in Table 3, there was a significant difference between ED + and ED– when comparing the GQ7 items (*p* < 0.001), GQ8 items (p < 0.001) and GQ loss of control (item 8) (*p* < 0.001). The mean BED-Q load score for ED + was 15.09 (± 7.86) whereas ED– had a mean load score of 1.77 (± 3.45) (*p* < 0.001).

**Table 3:** Grazing and binge eating disorder (BED) symptomatology

Variables	ED+ N = 23 (17.56%)	ED− N = 108 (82.44%)	Mean difference [95% CI], *p*
BED-Q Load Score, mean (SD)	15.09 (± 7.86)	1.77 (± 3.45)	12.58 [10.53, 14.64] *p* < 0.001
BED-Q Load Score, *n* (%)			
0–9 = No BED symptoms to subclinical	6 (26.09%)	104 (96.30%)	
10–21 = Mild to moderate BED	12 (52.17%)	< 5	
22–35 = Difficult to extreme BED	5 (21.74%)	< 5	
GQ Score			
GQ7 items, mean (SD)	13.74 (± 6.70)	5.31 (± 5.00)	7.77 [5.36, 10.18] *p* < 0.001
GQ8 items, mean (SD)	15.74 (± 7.47)	6.22 (± 5.66)	8.81 [6.08, 11.54] *p* < 0.001
GQ loss of control (single question), mean (SD)	2.09 (± 1.20)	0.58 (± 1.00)	1.41 [0.93, 1.88] *p* < 0.001

* *The means of the groups are compared using linear regression adjusted for sex, age and BMI. ED = eating disorder, BED = binge eating disorder, BED-Q = binge eating disorder questionnaire, GQ = grazing questionnaire, SD = standard deviation, CI = confidence interval*

### Quality of life

There was a significant difference in the EQ-5D-5 L utility score (*p* < 0.001), mobility (*p* = 0.011), self-care (*p* = 0.004), usual activities (*p* = 0.010), pain/discomfort (*p* = 0.022), anxiety/depression (*p* = 0.004) and VAS score (*p* = 0.012) when comparing ED + with ED–. The results are shown in Table 4.

**Table 4:** EuroQol’s EQ-5D-5 L questionnaire

Variables	ED+ *N* = 22 (16.92%)	ED− *N* = 108 (83.08%)	Mean difference [95% CI], *p*
EQ-5D-5 L utility score, mean (SD)	0.52 (± 0.45)	0.79 (± 0.22)	−0.25 [−0.38, −0.13] *p* < 0.0001
Mobility, mean (SD)	2.14 (± 1.13)	1.61 (± 0.89)	0.55 [0.13, 0.97] *p* = 0.011
Self-care, mean (SD)	1.59 (± 1.10)	1.15 (± 0.38)	0.40 [0.13, 0.66] *p* = 0.004
Usual activities, mean (SD)	2.18 (± 1.26)	1.63 (± 0.83)	0.56 [0.14, 0.99] *p* = 0.010
Pain/discomfort, mean (SD)	2.82 (± 1.30)	2.31 (± 1.00)	0.58 [0.09, 1.07] *p* = 0.022
Anxiety/depression, mean (SD)	2.50 (± 1.41)	1.65 (± 0.90)	0.66 [0.22, 1–11] *p* = 0.004
VAS score, mean (SD)	47.41 (± 25.5)	61.05 (± 20.7)	−13.20 [−23.43, −2.98] *p* = 0.012

* *The means of the groups are compared using linear regression adjusted for sex, age and BMI. ED = eating disorder, SD = standard deviation, CI = confidence interval*

## Discussion

The aim of this study was to investigate the prevalence of EDs among patients referred to lifestyle courses at the Department of Health Promotion, Aalborg University Hospital, Denmark. Less than one-fifth of the patients met the criteria for an ED or ED subclinical symptoms using a self-reported questionnaire. A significant difference between both the EDE-Q global score and all four subscales was found when comparing ED + with ED−. Patients with ED + were more troubled by restraint, eating concern, shape concern and weight concern than ED−. Generally, ED− had a high EDE-Q score on the shape and weight concern subscales. This may, however, be expected when assessing a patient group with high weight. Although the figure of 7.35% with BED in this study is somewhat lower than in other studies, the ED + percentage in this study was comparable to that of bariatric surgery candidates, with a BED prevalence of 17% [10]; it is also in accordance with the estimated BED prevalence in individuals with higher body weight seeking help to lose weight, which is 13–27% [22]. As mentioned, patients participating in this study were not bariatric surgery candidates but a mixed group of patients referred from various somatic hospital departments.

No pattern was found when looking at referring departments. Thus, patients with an ED seemed to come from all the included somatic departments.

In the Department of Health Promotion, there is a subgroup of patients that does not seem to comply with treatment. The ED prevalence in this study could be a contributing factor that may explain why some patients do not respond to the intervention. Thus, one could hypothesize that the subgroup of patients with an ED may be in this group of non-responders simply because they receive a deficient treatment in this intervention approach. If this is the case, some patients are incorrectly treated, which could be harmful. Thus, this subgroup of patients suffering from an ED should be referred to specialized ED treatment rather than weight-focused intervention. This is important knowledge for clinics offering lifestyle courses for somatic outpatients with a focus on weight loss. Clinicians need to be aware that EDs are common in somatic departments among patients with higher body weight. Reinforcing or developing an ED is also a risk when offering a lifestyle course with a focus on weight loss. Thus, talking lifestyle and weight loss with patients is a very complex task, and it is important that clinicians are familiar with ED pathology and aware that EDs are common among somatic patients with higher body weight. Thus, increased knowledge about treatment and assessment of ED and focus on the need for referral to specialized ED treatment is substantial. Likewise, it could be important to introduce screening procedures to identify patients with EDs at an early stage during somatic hospital treatment in order to refer patients to specialized ED treatment when appropriate.

In a review by Conceição et al. (2014), a standardized definition on grazing was proposed. They concluded that studies considering loss of control as a core component of grazing suggested an association with increased psychopathological impairment, which may indicate that the core psychopathologic component is the sense of loss of control [23]. In this study, we included both the GQ7 and GQ8 for comparability, and when looking at both there was a significant difference between ED + and ED− (*p* < 0.001), indicating that ED + had a significantly higher grazing behaviour. Loss of control in ED + was significantly higher when looking at the GQ8 when compared to ED− (*p* < 0.001). Therefore, this study adds to the perception of loss of control as a core component of psychopathological impairment.

Patients in this study also had a poor QoL outcome compared to the general Danish population, where studies using the EQ-5D-5 L show a mean utility score of 0.90 (SD = 0.16) [24]. Both ED– (0.79, SD = 0.22) and ED+ (0.52, SD = 0.45) had a poorer QoL compared to the Danish population, but ED + also had a significantly poorer QoL compared to ED– (*p* < 0.001). Thus, ED + had a significantly lower score on all five dimensions and for the VAS score.

### Strengths and limitations

There are several strengths to this study. A satisfactory study sample was included and it should also be emphasized that the relatively large group of male participants included in this study is satisfactory as men are often underrepresented in studies on ED. Recruitment bias regarding the age of male participants and also gender was found. This may be because patients referred to the Department of Health Promotion are more likely to be female due to referral of patients from IVF and similar departments, but also because males contact the healthcare system later than females.

This study group is comparable to the distribution of patients in the somatic hospital. However, it should be emphasized that this group is not necessarily representative of the general population.

Use of the EDE-Q is another strength of the study as it is a validated and widely used tool for assessment of EDs both in ED populations and broad population samples. It is, however, a limitation that self-report questionnaires were applied rather than a diagnostic ED interview such as the Eating Disorder Examination. This would, however, not have been possible in this study setup. Another limitation is the use of the BED-Q, which is a completely new Danish instrument that has not yet been tested or validated. A further limitation that should be mentioned is that this, to the best of our knowledge, is the first time GQ has been applied on a Danish study sample. Therefore, Danish norms aren’t available for GQ yet. However, we used two independent translators, then consensus translated it, and finally it was back-translated by a third bilingual professional to achieve the highest translation standard as possible. To assess the reliability of the instruments used we could have calculated the Cronbach’s alpha coefficient on our population. Finally, it should be stressed as a limitation that the study cohort was composed of patients referred from various somatic departments, thus consisting of relatively small samples from each department. This underlines the importance of further research into the prevalence of EDs in somatic departments since it is poorly described in the current literature.

This study provides new information about the prevalence of EDs among patients in contact with the somatic healthcare system for a somatic disorder. Only a few participants were excluded from study and the high participation rate secured a robust study population. Therefore, the cohort may be representative of a large group of patients with higher weight who are in contact with the somatic healthcare system.

## Conclusion

Although the majority of individuals referred to a lifestyle course at the Department of Health Promotion do not suffer from an ED, the prevalence of EDs or subclinical ED is notable. Furthermore, a large proportion of patients with an ED had grazing behaviour accompanied by loss of control. Overall, patients with an ED or subclinical ED symptoms had poor QoL compared to patients without ED symptomatology. Thus, the ED prevalence among patients within somatic hospital departments may be substantial, which underlines the importance of further screening and research into EDs among patients in various somatic hospital settings.

Further research should investigate ED prevalence in somatic patient populations, e.g., diabetes type II and sleep apnea, as these findings suggest that ED may be severely underdiagnosed. Furthermore, clinicians in somatic specialties should assess for EDs when treating patients presenting with symptoms that meet the criteria rather than solely focusing on weight and lifestyle.

## Data Availability

The datasets generated during and/or analyzed during the current study are available from the corresponding author on reasonable request.

## References

[1] Treasure J, Claudino AM, Zucker N (2010). Eating disorders. Lancet (London England).

[2] Whiteford HA, Degenhardt L, Rehm J (2013). Global burden of Disease attributable to mental and substance use disorders: findings from the global burden of Disease Study 2010. Lancet (London England).

[3] Dingemans AE, van Furth EF (2012). Binge eating disorder psychopathology in normal weight and obese individuals. Int J Eat Disord.

[4] McCuen-Wurst C, Ruggieri M, Allison KC (2018). Disordered eating and obesity: associations between binge eating-disorder, night-eating syndrome, and weight-related co-morbidities. Ann N Y Acad Sci.

[5] Goodpaster K, Marek R, Lavery M, et al. Graze eating among bariatric Surgery candidates: Prevalence and Psychosocial correlates. Surg Obes Relat Dis. 2016;12. 10.1016/j.soard.2016.01.006. 10.1016/j.soard.2016.01.006 27134201

[6] Heriseanu AI, Hay P, Touyz S (2019). Grazing behaviour and associations with obesity, eating disorders, and health-related quality of life in the Australian population. Appetite.

[7] Reas DL, Lindvall Dahlgren C, Wonderlich J (2019). Confirmatory factor analysis and psychometric properties of the Norwegian version of the repetitive eating questionnaire: further evidence for two distinct subtypes of grazing behaviour. Eur Eat Disord Rev J Eat Disord Assoc.

[8] Kessler RC, Berglund PA, Chiu WT (2013). The prevalence and correlates of binge eating disorder in the World Health Organization World Mental Health Surveys. Biol Psychiatry.

[9] Micali N, Martini MG, Thomas JJ (2017). Lifetime and 12-month prevalence of eating disorders amongst women in mid-life: a population-based study of diagnoses and risk factors. BMC Med.

[10] Dawes AJ, Maggard-Gibbons M, Maher AR (2016). Mental Health conditions among patients seeking and undergoing bariatric Surgery: a Meta-analysis. JAMA.

[11] Harris PA, Taylor R, Thielke R (2009). Research electronic data capture (REDCap)-A metadata-driven methodology and workflow process for providing translational research informatics support. J Biomed Inform.

[12] Harris PA, Taylor R, Minor BL (2019). The REDCap consortium: building an international community of software platform partners. J Biomed Inform.

[13] Gideon N, Hawkes N, Mond J (2016). Development and psychometric validation of the EDE-QS, a 12 item short form of the eating disorder examination questionnaire (EDE-Q). PLoS ONE.

[14] Fairburn CG, Beglin SJ (1994). Assessment of eating disorders: interview or self-report questionnaire?. Int J Eat Disord.

[15] Poulsen S, Lunn S, Daniel SIF (2014). A randomized controlled trial of psychoanalytic psychotherapy or cognitive-behavioral therapy for bulimia nervosa. Am J Psychiatry.

[16] Lichtenstein MB, Haastrup L, Johansen KK, et al. Validation of the eating disorder examination questionnaire in Danish eating disorder patients and athletes. J Clin Med. 2021;10. 10.3390/jcm10173976. 10.3390/jcm10173976 PMC8432050 34501422

[17] Lane B, Szabó M (2013). Uncontrolled, Repetitive Eating of Small Amounts of Food or ‘Grazing’: development and evaluation of a New measure of atypical eating. Behav Chang.

[18] Herdman M, Gudex C, Lloyd A (2011). Development and preliminary testing of the new five-level version of EQ-5D (EQ-5D-5L). Qual life Res an Int J Qual life Asp Treat care Rehabil.

[19] Feng YS, Kohlmann T, Janssen MF, Buchholz I (2021). Psychometric properties of the EQ-5D-5L: a systematic review of the literature. Qual Life Res.

[20] Jensen ES, Linnet J, Holmberg TT (2020). Effectiveness of internet-based guided self-help for binge-eating disorder and characteristics of completers versus noncompleters. Int J Eat Disord.

[21] World Health Organization A healthy lifestyle - WHO recommendationso Title. https://www.who.int/europe/news-room/fact-sheets/item/a-healthy-lifestyle---who-recommendations. Accessed 24 Apr 2023.

[22] Montano CB, Rasgon NL, Herman BK (2016). Diagnosing binge eating disorder in a primary care setting. Postgrad Med.

[23] Conceição EM, Mitchell JE, Engel SG (2014). What is grazing? Reviewing its definition, frequency, clinical characteristics, and impact on bariatric Surgery outcomes, and proposing a standardized definition. Surg Obes Relat Dis off J Am Soc Bariatr Surg.

[24] Jensen MB, Jensen CE, Gudex C (2021). Danish population health measured by the EQ-5D-5L. Scand J Public Health.

